# TECHNOLOGY AND GROUP EXERCISE INTERVENTIONS FOR PEOPLE WITH DEMENTIA OR MILD COGNITIVE IMPAIRMENT: A SCOPING REVIEW

**DOI:** 10.1093/geroni/igac059.271

**Published:** 2022-12-20

**Authors:** Hannah Levine, Paavan Randhawa, JuYoung Park, Lillian Hung

**Affiliations:** Florida Atlantic University, Charles E. Schmidt College of Medicine, Boca Raton, Florida, United States; Langara College, Vancouver, British Columbia, Canada; Florida Atlantic University College of Social Work and Criminal Justice, Boca Raton, Florida, United States; University of British Columbia, Vancouver, British Columbia, Canada

## Abstract

We conducted a scoping review to analyze evidence about online group-based exercise programs. We searched six electronic databases and conducted team analysis with patient and family partners. Of 1,166 screened articles, the final review included 8 publications. Results identified three types of technology-based group exercise interventions for people with dementia or mild cognitive impairment (MCI): (a) exergames, (b) virtual cycling, and (c) video-conferencing platforms. These studies used psychosocial, physical function, biomarker, and cognitive outcome measures. The review identified three key impacts: (a) feasibility and accessibility; (b) physical, psychosocial, and cognitive benefits; and (c) adaptations necessary for persons with dementia or MCI. Over all, technology-based group exercise interventions were found to be accessible, feasible, and acceptable to persons with dementia or MCI. However, a “one-size-fits-all” game approach often did not work, suggesting that exercise interventions should be adaptable to meet the various needs of individual participants.

